# Association of the triglyceride-glucose index with the severity and short-term prognosis of Guillain-Barré syndrome

**DOI:** 10.3389/fimmu.2026.1813152

**Published:** 2026-06-08

**Authors:** Lijuan Wang, Xiang Li, Zikai Zhou, Yuxi Tian, Siwen Li, Wencan Jiang, Kelin Chen, Yuxin Chen, Jie Liu, Guanghui Zheng, Xin gao Wang, Guojun Zhang

**Affiliations:** 1 Department of Clinical Diagnosis, Laboratory of Beijing Tiantan Hospital, Capital Medical University, Beijing, China; 2 Laboratory Diagnosis Platform for Nervous System Infectious Diseases, Laboratory for Clinical Medicine, Capital Medical University, Beijing, China; 3 Beijing Key Laboratory of Biomarker Screening and Translational Research in Neurological Diseases, Beijing, China; 4 Department of Neurology, Beijing Tiantan Hospital, Capital Medical University, Beijing, China

**Keywords:** binary logistic regression analysis, Guillain-Barré syndrome, receiver operating characteristic curves, restricted cubic spline, triglyceride-glucose index

## Abstract

**Background:**

Guillain-Barré syndrome (GBS) is a peripheral nerve inflammatory disorder driven by immune-mediated mechanisms. Currently, there is a lack of research investigating the association between the triglyceride-glucose (TyG) index and GBS, and this study sought to explore their possible relationship.

**Methods:**

203 patients with GBS from Beijing Tiantan Hospital were retrospectively enrolled in this study. The relationship of the TyG index with severe and short-term poor prognosis in GBS patients was investigated using least absolute shrinkage and selection operator (LASSO) regression, binary logistic regression, restricted cubic spline (RCS) analysis and receiver operating characteristic (ROC) curve.

**Results:**

The TyG index (OR: 2.77; 95% CI: [1.51-5.30]; P = 0.001) was identified as independent risk factors for severe GBS. Conversely, albumin (ALB) (OR: 0.89; 95% CI: [0.82-0.96]; P = 0.006) and the Medical Research Council (MRC) sum score (OR: 0.93; 95% CI: [0.90-0.96]; P<0.001) acted as protective factors. Additionally, the TyG index (OR: 1.85; 95% CI: [1.05-3.32]; P = 0.036) emerged as an independent risk factor for poor short-term prognosis, whereas the MRC sum score (OR: 0.94; 95% CI: [0.91-0.96]; P<0.001) was recognized as a protective factor. Furthermore, TyG index showed a linear correlation with severe GBS (overall P<0.01, nonlinear P = 0.112) and short-term poor prognosis in GBS patients (overall P = 0.068, nonlinear P = 0.277). Used alone, the TyG index yielded area under the curve (AUC) values of 0.644 and 0.589 for predicting severe GBS and short-term poor-prognosis GBS, respectively. When combined with conventional clinical markers, the model achieved an AUC of 0.808 (sensitivity 0.732, specificity 0.776) for severe GBS and 0.765 (sensitivity 0.763, specificity 0.701) for short-term poor prognosis. Bootstrap internal validation showed optimism bias/corrected AUC of 0.008/0.800 and 0.006/0.759 for the two models. After incorporating the TyG index into the baseline model, the net reclassification index (NRI) was significantly elevated, while the integrated discrimination improvement (IDI) showed a slight improvement (all P<0.05).

**Conclusions:**

Elevated TyG index is independently linked to higher risks of severe phenotype and short-term poor prognosis in GBS patients. Combined with conventional clinical indicators, TyG index may help predict severe cases and adverse short-term prognosis in GBS.

## 1 Introduction

Guillain-Barré syndrome (GBS) is an acute, immune-mediated disorder characterized by the rapid onset of muscle weakness and areflexia, which typically develops subsequent to an infectious trigger, most commonly viral or bacterial pathogens, such as Campylobacter jejuni and Epstein-Barr virus and so on (1, 2). GBS severity varies significantly, ranging from mild symptoms to severe forms that result in paralysis or respiratory failure, which thus necessitates urgent medical intervention. Despite substantial improvements in GBS patient outcomes with early diagnosis and intervention, particularly with Intravenous Immunoglobulin (IVIg) or plasmapheresis (3, 4), the disorder continues to be linked with notable morbidity and mortality. Currently, despite the evolving understanding of the underlying mechanisms driving GBS and its clinical outcomes, early identification of patients at risk for severe progression or poor prognosis remains a crucial challenge. Identifying factors predictive of disease severity and short-term prognosis is crucial for improving clinical management and optimizing patient outcomes.

Insulin resistance (IR) refers to the reduced responsiveness of target tissues to normal circulating concentrations of insulin. This results in ineffective glucose uptake by target cells, leading to subsequent emergence of metabolic irregularities (5). Initially introduced in 2008, the triglyceride-glucose (TyG) index represents a composite measure that comprises fasting triglyceride (TG) and fasting plasma glucose (FPG) levels (6). While the hyperinsulinemic-euglycemic clamp test is the established gold standard for assessing IR, the TyG index has demonstrated enhanced sensitivity and specificity in its identification (7). Consequently, the TyG index can function as an alternative biomarker for IR owing to its straightforward calculation and minimal time or cost constraints.

Numerous high-quality clinical studies have underscored the association of the TyG index with a variety of diseases, including diabetes (8, 9), cardiovascular disease (10, 11), fatty liver disease (12, 13), kidney disease (14, 15), and reproductive system disorders (16, 17). Beyond these conditions, the significance of the TyG index in the context of neurological disorders is becoming increasingly pronounced. Cross-sectional studies have shown that individuals with dementia or mild cognitive impairment exhibit higher TyG index levels compared to those with normal cognitive function. Furthermore, the TyG index is independently associated with cognitive impairment in patients with Parkinson’s disease, and this association remains significant even after excluding individuals with diabetes (18). Data from 410,515 individuals in the UK Biobank have revealed a nonlinear relationship between the TyG index (and its associated measures) and mortality attributable to neurological disorders (19). In the context of cerebrovascular diseases, the TyG index not only serves as a predictor for the incidence and recurrence of stroke but is also independently associated with early neurological deterioration following intravenous thrombolysis or mechanical thrombectomy, as well as unfavorable outcomes at the three-month follow-up (20). Moreover, additional research has demonstrated a nonlinear relationship between the TyG index and the risk of developing ICU delirium: when the TyG index exceeds 8.912, the risk of ICU delirium increases significantly, which confirms the TyG index as an independent predictor of acute brain dysfunction (21).

Nonetheless, the role of the TyG index in GBS is still inadequately elucidated. To this end, the present study was carried out to explore the relationship between the TyG index and the severity of GBS, along with its potential significance in forecasting short-term outcomes. Insight into this association could allow for early identification of high-risk patients by clinicians, thereby informing more personalized and effective treatment approaches to optimize GBS outcomes.

## 2 Materials and methods

### 2.1 Subjects

A total of 203 patients diagnosed with GBS were admitted to Beijing Tiantan Hospital, Capital Medical University between January 2015 and April 2026 and included in the study cohort. All patients fulfilled the diagnostic criteria for GBS as outlined by the European Neurological Society (22). The inclusion criteria were as follows: 1) Patients must exhibit gradually worsening, symmetric limb weakness, typically starting in the lower limbs and progressing upwards; 2) Accompanied by diminished or absent deep tendon reflexes; 3) Disease progression must not exceed 4 weeks; 4) Clinical symptoms must align with the characteristics of acute immune-mediated disorders, without evidence of central nervous system injury or underlying systemic diseases. The exclusion criteria included: 1) Clear asymmetrical weakness or hyperreflexia; 2) Primary symptoms of sensory disturbances or persistent pain; 3) Altered consciousness or other central nervous system symptoms associated with fever; 4) Significant pleocytosis in cerebrospinal fluid, indicating infectious or inflammatory pathology. Informed consent was obtained from each participant. This study received approval from the Ethics Committee of Beijing Tiantan Hospital, Capital Medical University (KY-2022-039-01).

### 2.2 Data collection

Following a 15-minute seated rest period, diastolic and systolic blood pressure readings were recorded from the right arm using a standard mercury sphygmomanometer. Peripheral blood samples were obtained in the morning following a 12-hour fasting period. Body mass index (BMI) was calculated with the formula: weight (kg)/height (m²). Peripheral venous blood was analyzed to evaluate various parameters, including white blood cell count (WBC), platelet count (PLT), albumin (ALB), triglycerides (TG), total cholesterol (CHO), high-density lipoprotein (HDL) cholesterol, low-density lipoprotein (LDL) cholesterol, Apo lipoprotein A1 (APOA1), Apo lipoprotein B (APOB), and fasting glucose (GLU). The TyG index was determined following the formula: TyG index=Ln [fasting triglyceride (mg/dL) * fasting glucose (mg/dL)/2]. In our laboratory, glucose and triglyceride measurements are reported in mmol/L, while the TyG calculation formula uses mg/dL, necessitating unit conversions. Specifically, 1 mmol/L of glucose is equivalent to 18.02 mg/dL, and 1 mmol/L of triglycerides corresponds to 88.54 mg/dL. The laboratory data utilized in this study were obtained from routine clinical tests. We ensured the accuracy of these measurements through regular instrument maintenance, internal quality control, and participation in external proficiency testing.

### 2.3 Evaluation of disease severity and short-term prognosis in GBS

The clinical scores of the 203 GBS patients were assessed at admission and discharge using the extensively accepted Hughes Functional Grading Scale (HFGS) (22, 23), a specifically designed tool for evaluating disability in GBS patients. The grading scale was categorized as: 0, no symptoms; 1, mild symptoms with the capability to run; 2, ability to walk independently for 10 meters or more (unable to run); 3, ability to walk 10 meters with assistance; 4, bedridden or wheelchair-dependent; 5, requiring supplemental ventilation for at least part of the day; and 6, deceased. GBS was stratified into severe and mild subtypes based on admission disability scores: severe GBS was assigned to scores ≥3, and mild GBS to scores <3. A GBS disability score of ≥3 at discharge was indicative of poor short-term prognosis, with a score of <3 denoting a good short-term prognosis (24).

### 2.4 Assessment of muscle strength in GBS

The Medical Research Council (MRC) scale (25), initially established in 1943 and later refined by Kleyweg et al. during their 1988 clinical trial on GBS, serves as a standardized tool for evaluating muscle strength of GBS patients. Herein, the MRC scale examined the strength of 12 muscle groups, which included both the left and right sides of the body. These groups comprised shoulder abduction, elbow flexion, wrist extension, hip flexion, knee extension, and ankle dorsiflexion. Each muscle group was assigned a rating on a scale from 0 to 5, based on the following criteria: 0, no contraction; 1, palpable contraction without visible joint movement; 2, movement possible in a horizontal plane but insufficient to overcome gravity; 3, horizontal plane movement (unable to overcome gravity); 4, ability to resist some resistance; and 5, normal strength. Total scores range from 0 to 60, where lower scores correspond to reduced muscle strength. This evaluation method is extensively utilized in both clinical and research settings to gauge GBS severity and track patient outcomes.

### 2.5 Statistics

All statistical analyses and graphical plotting were performed using R software (version 4.5.2). Quantitative data conforming to normal distribution were presented as mean ± standard deviation (SD) and compared via the independent sample t-test. Non-normally distributed quantitative data were expressed as median (interquartile range, Q1–Q3) and analyzed using the Wilcoxon rank-sum test. Categorical variables were described as counts (percentages) and compared with the chi-square test.

Least absolute shrinkage and selection operator (LASSO) regression was used to screen potential variables associated with severe GBS and poor short-term prognosis of GBS. The penalty parameter λ of LASSO regression was determined by 10-fold cross-validation, and the optimal λ was selected according to the one-standard-error (1-SE) rule. All participants were stratified into four subgroups according to TyG index quartiles. Baseline clinical and laboratory indicators among subgroups were compared, and the trend of adverse clinical outcomes was assessed using the Cochran-Armitage trend chi-square test. Restricted cubic spline (RCS) regression was further applied to characterize the dose-response relationship between the TyG index and study endpoints.

Receiver operating characteristic (ROC) curves were plotted to calculate the area under the curve (AUC), sensitivity and specificity of the predictive model. The DeLong test was used for pairwise comparison of AUCs between different models. Net reclassification improvement (NRI) and integrated discrimination improvement (IDI) were calculated to evaluate the incremental predictive value of the TyG index. Bootstrap resampling was adopted for internal validation of model stability. A two-tailed P value < 0.05 was defined as statistically significant.

## 3 Results

### 3.1 Demographic and clinical characteristics in patients with GBS

A total of 203 patients diagnosed with GBS were consecutively enrolled in the present study. Among these participants, 127 had favorable short-term prognoses and 76 had poor short-term prognoses; 127 patients were classified as severe cases and 76 as mild cases. Clinical characteristics stratified by disease severity and short-term prognosis are summarized in **Tables 1** and **2**, respectively. Compared with mild GBS patients, those with severe disease had lower admission MRC sum score, HDL and ALB levels, along with higher TyG index and TG levels (all P<0.05). Similarly, patients with unfavorable short-term prognosis presented decreased MRC sum score and ALB concentrations at admission (all P<0.05). No significant differences were observed in other baseline characteristics across both comparison groups.

**Table 1:** Clinical and laboratory characteristics in mild and severe GBS.

Characteristic	Severe	Mild	*P* value
	N=127	N=76	
Age, y	51.00 (39.00, 60.00)	48.00 (33.50, 61.00)	0.167
Sex, female, n (%)	65 (51.2%)	46 (60.5%)	0.196
SBP, mmHg	136.39 ± 17.34	135.13 ± 12.95	0.556
DBP, mmHg	85.55 ± 10.87	84.92 ± 9.06	0.657
BMI, kg/m^2^	24.91 (22.68, 27.20)	23.91 (21.48, 26.43)	0.155
MRC sum score	40.00 (30.00, 50.00)	56.00 (47.50, 60.00)	<0.001
TyG index	8.71 ± 0.56	8.46 ± 0.52	0.002
APOB, g/L	0.82 (0.72, 0.96)	0.85 (0.72, 0.96)	0.806
Triglyceride, mmol/L	1.55 (1.02, 2.04)	1.14 (0.92, 1.50)	<0.001
Cholesterol, mmol/L	4.09 (3.56, 4.51)	4.02 (3.51, 4.55)	0.615
LDL, mmol/L	2.49 ± 0.76	2.58 ± 0.76	0.465
APOA1, g/L	1.18 (1.02, 1.29)	1.15 (1.08, 1.35))	0.510
HDL, mmol/L	1.06 (0.94, 1.21)	1.15 (0.99, 1.26)	0.037
Albumin, g/L	36.90 (34.55, 39.25)	39.20 (37.05, 41.45)	<0.001
WBC count, 10^9^/L	7.32 (5.74, 9.00)	6.65 (5.64, 8.00)	0.086
Platelet count, 10^9^/L	253.00 (214.50, 311.00)	236.50 (203.75, 290.00)	0.138
Glucose, mmol/L	4.91 (4.33, 5.70)	4.79 (4.36, 5.21)	0.427
Hypertension, n (%)	46 (36.2%)	20 (26.3%)	0.145
Diabetes mellitus, n (%)	19 (15.0%)	9 (11.8%)	0.533
Heart disease, n (%)	7 (5.5%)	1 (1.3%)	0.263
Cigarette smoking, n (%)	34 (26.8%)	21 (27.6%)	0.894
Alcohol consumption, n (%)	27 (21.3%)	22 (28.9%)	0.215
Preceding infection, n (%)	85 (66.9%)	49 (64.5%)	0.721
Surgery, n (%)	22 (17.3%)	9 (11.8%)	0.293
Trauma, n (%)	20 (15.7%)	12 (15.8%)	0.994

Data presented as mean ± SD, median (Q1-Q3) or percentage. GBS, Guillain-Barré syndrome; SBP, systolic blood pressure; DBP, diastolic blood pressure; BMI, Body mass index; MRC, Medical Research Council ; TyG index, triglyceride-glucose index; APOB, apolipoprotein B; LDL, low-density lipoprotein; APOA1, apolipoprotein A1; HDL, high-density lipoprotein; WBC, White Blood Cell.

**Table 2:** Clinical and laboratory characteristics in GBS with good and poor short-term prognosis.

Characteristic	Good prognosis	Poor prognosis	*P* value
	N=127	N=76	
Age, y	49.00 (34.50, 60.00)	52.50 (40.00, 61.00)	0.156
Sex, female, n (%)	72 (56.7%)	39 (51.3%)	0.456
SBP, mmHg	134.60 ± 14.06	138.13 ± 18.28	0.15
DBP, mmHg	85.02 ± 9.55	85.82 ± 11.27	0.606
BMI, kg/m^2^	24.46 (22.33, 27.04)	24.80 (22.17, 27.09)	0.574
MRC sum score	50.00 (42.00, 60.00)	36.00 (26.25, 45.75)	<0.001
TyG index	8.56 ± 0.52	8.71 ± 0.61	0.076
APOB, g/L	0.84 (0.73, 0.95)	0.82 (0.72, 0.98)	0.973
Triglyceride, mmol/L	1.26 (0.95, 1.78)	1.61 (1.01, 2.04)	0.068
Cholesterol, mmol/L	4.00 (3.50, 4.51)	4.16 (3.60, 4.62)	0.161
LDL, mmol/L	2.50 (1.94, 3.00)	2.45 (2.03, 2.98)	0.696
APOA1, g/L	1.15 (1.04, 1.30)	1.20 (1.05, 1.33)	0.305
HDL, mmol/L	1.08 (0.96, 1.25)	1.10 (0.95, 1.24)	0.883
Albumin, g/L	38.50 (35.80, 40.65)	36.90 (34.88, 39.23)	0.008
WBC count, 10^9^/L	6.75 (5.60, 8.34)	7.51 (5.96, 9.10)	0.099
Platelet count, 10^9^/L	248.00 (213.00, 299.00)	245.50 (198.00, 310.25)	0.932
Glucose, mmol/L	4.82 (4.30, 5.50)	4.95 (4.45, 5.63)	0.281
Hypertension, n (%)	38 (29.9%)	28 (36.8%)	0.308
Diabetes mellitus, n (%)	18 (14.2%)	10 (13.2%)	0.839
Heart disease, n (%)	5 (3.9%)	3 (3.9%)	1
Cigarette smoking, n (%)	34 (26.8%)	21 (27.6%)	0.894
Alcohol consumption, n (%)	32 (25.2%)	17 (22.4%)	0.649
Preceding infection, n (%)	83 (65.4%)	51 (67.1%)	0.799
Surgery, n (%)	19 (15.0%)	12 (15.8%)	0.874
Trauma, n (%)	20 (15.7%)	12 (15.8%)	0.994

Data presented as mean ± SD, median (Q1-Q3) or percentage. GBS, Guillain-Barré syndrome; SBP, systolic blood pressure; DBP, diastolic blood pressure; BMI, Body mass index; MRC, Medical Research Council ; TyG index, triglyceride-glucose index; APOB, apolipoprotein B; LDL, low-density lipoprotein; APOA1, apolipoprotein A1; HDL, high-density lipoprotein; WBC, White Blood Cell.

### 3.2 Determination of variables included in multivariate regression model

**Figure 1:**
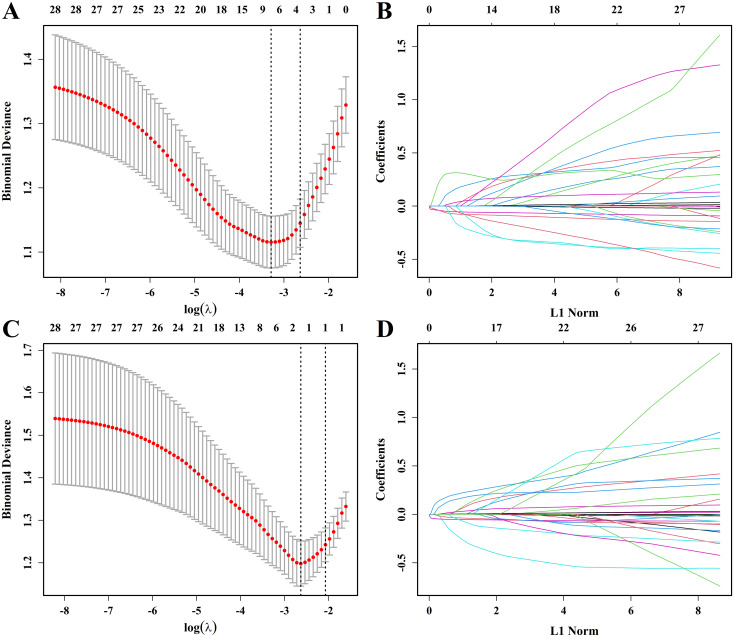
LASSO-based variable selection for severe GBS and poor short-term prognosis. **(A, B)** LASSO results for severe outcomes; **(C, D)** LASSO results for poor short-term prognosis. **(A, C)** Ten-fold cross-validation curves showing binomial deviance across a range of penalty parameters (λ). The vertical dashed lines indicate the optimal λ values determined by the minimum criterion (λ_min_) and the one-standard-error rule (λ_1se_). The numbers above the plots denote the number of non-zero coefficients for each λ. **(B, D)** LASSO coefficient profiles of all candidate variables plotted against the L1 norm, illustrating the shrinkage and selection process of regression coefficients.

The binomial deviance was plotted as a function of log(λ), where λ denotes the tuning hyperparameter. Vertical dotted lines indicate the optimal λ values determined by the minimum and 1-SE criteria. Based on 10-fold cross-validation, the optimal λ was selected using the 1-SE criterion. For severe GBS, the optimal λ was 0.07158125 (log(λ) =-2.636922115; **Figure 1**), while for GBS with poor short-term prognosis, it was 0.125903485 (log(λ) =-2.072239655; **Figure 1**). Corresponding LASSO coefficient profiles, plotted against the L1 norm, are shown in **Figure 1** (severe GBS) and **Figure 1** (poor short-term prognosis). As shown in **Table 3**, three variables including MRC sum score, ALB, and TyG index were screened for severe GBS, with corresponding regression coefficients of -0.03875309, -0.036532331, and 0.206855052, respectively. For patients with poor short-term prognosis, only the MRC sum score entered the final model, with a regression coefficient of -0.020919946. Given that the TyG index was the primary focus of this study; both the TyG index and MRC sum score were included in the multivariate regression model for predicting poor short-term outcomes. As summarized in **Supplementary Table 1**, the variance inflation factor (VIF) for all included variables were less than 2, confirming no obvious multicollinearity among variables.

**Table 3:** Variables screened via LASSO regression based on λ_1_se.

Outcome	Variable	Coefficient (λ_1_se)	Selected at λ_1_se
Severe GBS	MRC sum score	-0.03875309	Yes
	Albumin	-0.036532331	Yes
	TyG index	0.206855052	Yes
GBS with Poor short-term prognosis	MRC sum score	-0.020919946	Yes

λ_1_se, the optimal penalty parameter determined via 10-fold cross-validation and selected according to the one-standard-error (1-SE) rule in LASSO regression.

### 3.3 Correlation of TyG with severe and poor short-term prognosis GBS

**Figure 2:**
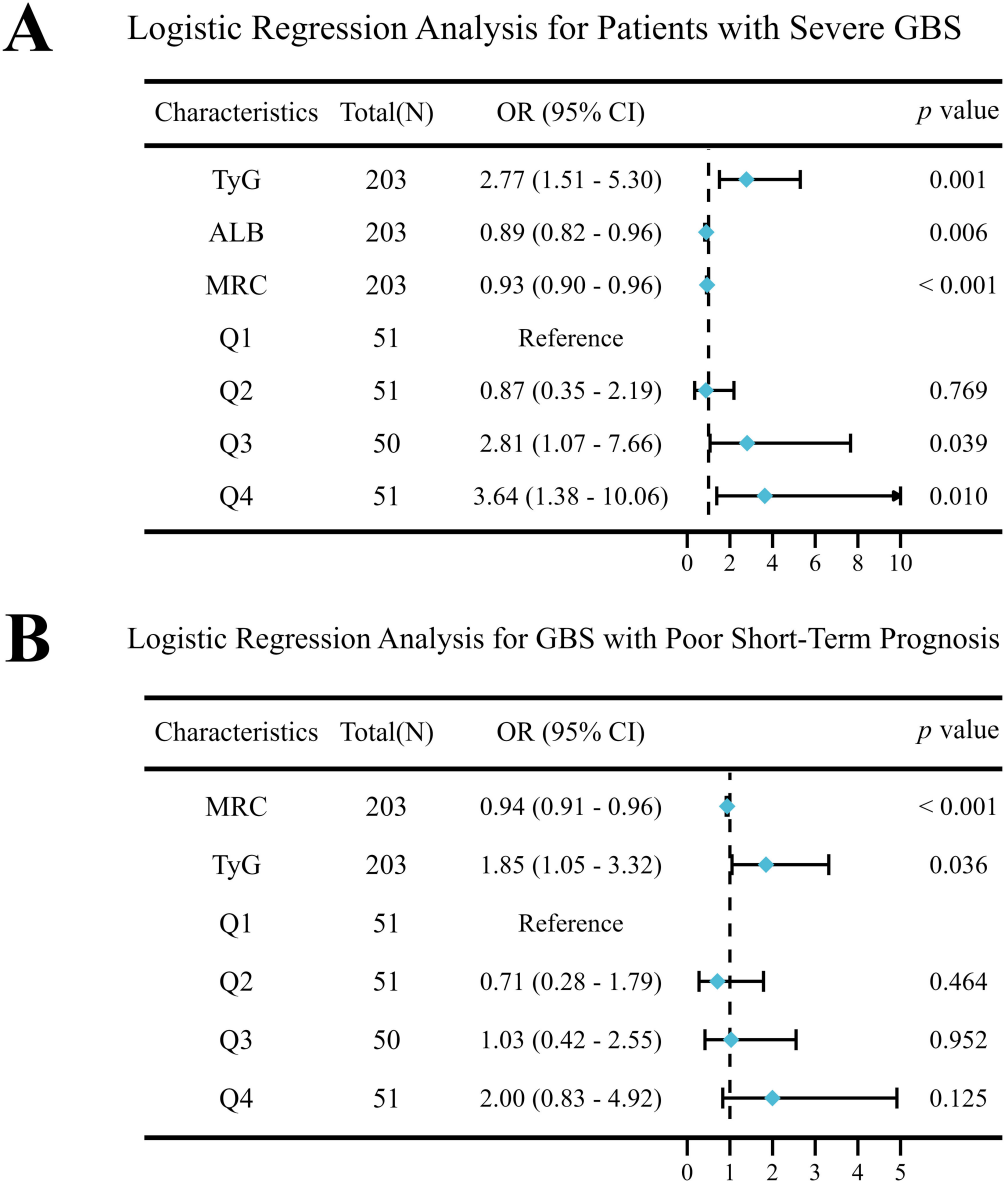
Forest plots depicting the risk of severe GBS and poor short-term prognosis. **(A)** The TyG index was identified as independent risk factors for severe GBS. In contrast, ALB and the MRC sum score functioned as protective factors. The risk of severe GBS associated with TyG index Q3/Q4 was 2.81/3.64 times greater than that of Q1. **(B)** The TyG index served as a risk factor for poor short-term prognosis in GBS, with no significant differences in prognostic outcomes observed across the various quartiles. The MRC sum score was considered a protective factor.

Multivariate binary logistic regression analysis was performed to identify independent risk factors for the two study endpoints. In the severe GBS model (**Figure 2**), an elevated TyG index was identified as an independent risk factor for severe GBS (OR = 2.77, 95% CI: 1.51–5.30, P = 0.001). By contrast, ALB (OR = 0.89, 95% CI: 0.82–0.96, P = 0.006) and MRC sum score (OR = 0.93, 95% CI: 0.90–0.96, P<0.001) acted as independent protective factors. Compared with patients in the Q1 quartile of the TyG index, those in Q3 and Q4 exhibited a significantly higher risk of severe GBS (all P<0.05). In the poor short-term prognosis model (**Figure 2**), a higher TyG index independently increased the risk of unfavorable short-term outcomes (OR = 1.85, 95% CI: 1.05–3.32, P = 0.036). Meanwhile, MRC sum score served as an independent protective factor against poor prognosis (OR = 0.94, 95% CI: 0.91–0.96, P<0.001). No significant differences in the risk of adverse short-term outcomes were detected across TyG index quartiles (all P>0.05).

**Figure 3:**
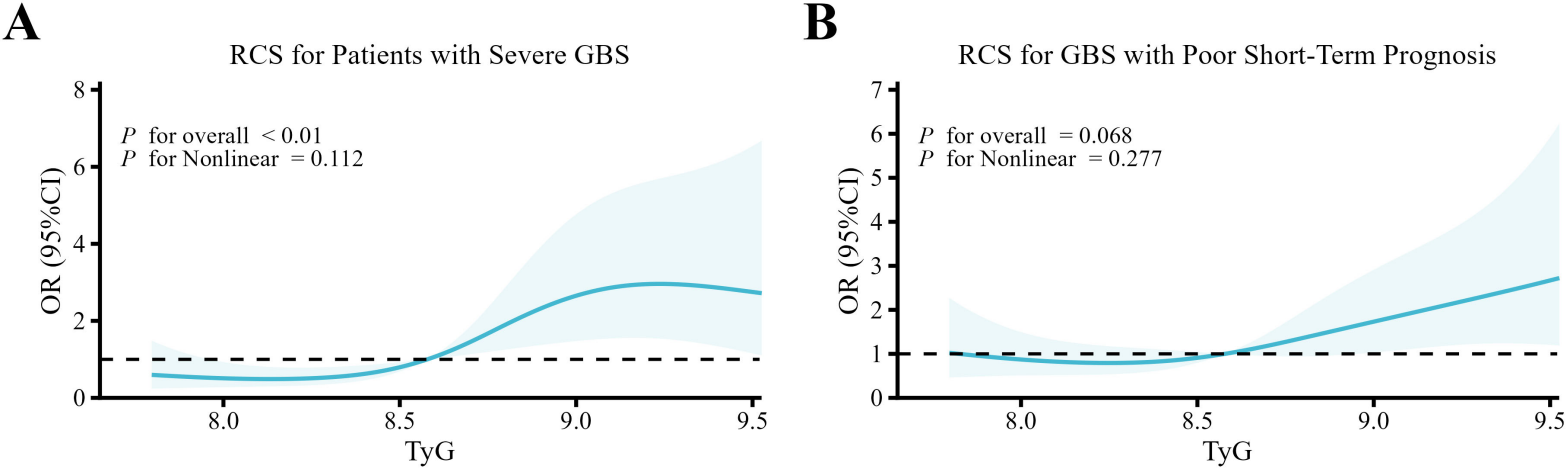
Restricted cubic spline fitting for the association between the TyG index and GBS. Association of the TyG index with severe and poor short-term prognosis GBS by RCS analysis. **(A)** For severe GBS, the overall association was statistically significant (P<0.01), while no significant nonlinear trend was observed (P for nonlinear = 0.112). **(B)** For poor short-term prognosis GBS, the overall association showed a borderline statistical significance (P = 0.068), with no obvious nonlinear correlation (P for nonlinear = 0.277).

RCS analysis showed that for severe GBS (**Figure 3**), the overall association between the TyG index and disease risk was statistically significant (P for overall < 0.01), whereas no significant nonlinear trend was found (P for nonlinear = 0.112). The risk of severe GBS increased linearly with rising TyG index. For poor short-term prognosis (**Figure 3**), the overall association reached borderline statistical significance (P for overall = 0.068), with no evident nonlinear relationship (P for nonlinear = 0.277).

### 3.4 Characteristics of GBS patients based on the quartile of the TyG index

**Figure 4:**
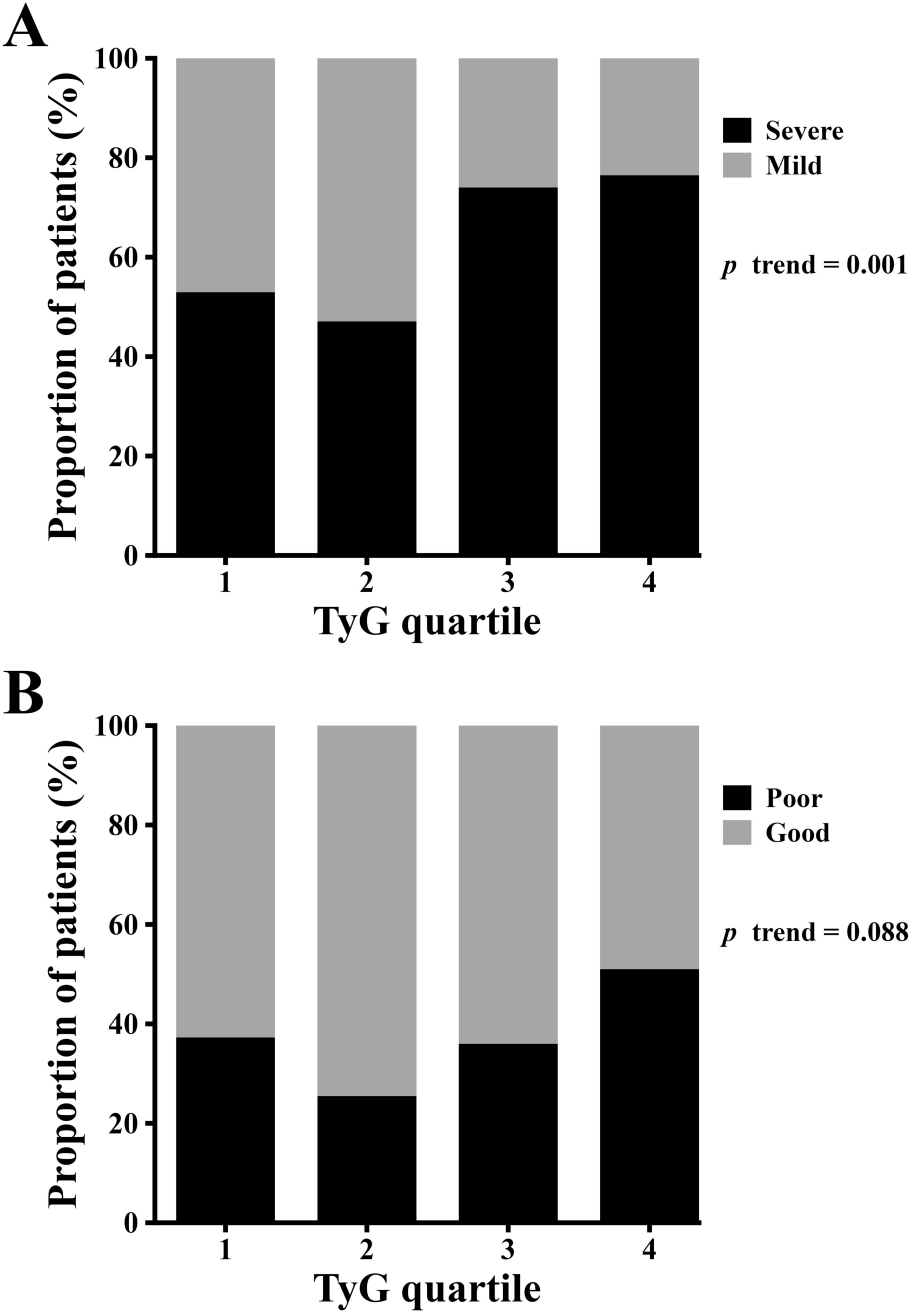
Proportional distribution of severe and poor short-term prognosis GBS. The TyG index was set according to the quartile (Q) levels of the enrolled GBS patients. **(A)** The prevalence of severe GBS appeared to rise as the TyG index increased (P = 0.001). **(B)** The proportion of patients with a poor short-term prognosis for GBS showed no significant change with increasing TyG index (P = 0.088).

For baseline characterization of GBS patients by TyG index, subjects were divided into four quartiles: Q1: <8.24, Q2: 8.24-8.58, Q3: 8.58-8.96, and Q4: >8.96. TyG quartile-stratified GBS patient characteristics are presented in **Table 4**, with a notable trend of increasing age in patients with higher TyG index. The quartiles of the TyG index demonstrated a positive correlation with GBS disability scores at both admission and discharge, as well as with SBP, DBP, BMI, total cholesterol levels, and incidence of hypertension and diabetes mellitus. Conversely, a negative correlation was observed between TyG quartiles and HDL levels (all P<0.05). As TyG index increased, the proportion of patients with severe GBS gradually rose (**Figure 4**, P*=*0.001); the proportion of patients with poor short-term prognosis showed an upward trend without statistical significance (**Figure 4**, P = 0.088).

**Table 4:** Characteristics of patients with GBS according to TyG index quartile.

Characteristics	Q1 (<8.24)	Q2 (8.24-8.58)	Q3 (8.58-8.96)	Q4 (>8.96)	*p* Trend
	N=51	N=51	N=50	N=51	
Sex, female, n (%)	24 (47.1)	23 (45.1)	20 (40.0)	25 (49.0)	0.977
Age, y	39 (30, 57.5)	51 (34, 60.5)	51.5 (41.5, 59)	57 (42.5, 62)	0.003
GBS disability score at admission	3 (2, 4)	2 (2, 3)	3 (2.25, 4)	4 (3, 4)	0.037
GBS disability score at discharge	2 (1, 3)	2 (1, 2.5)	2 (2, 3)	3 (2, 4)	0.024
SBP, mmHg	132.61±15.18	133.18±16.44	137.42±14.73	140.51±15.97	0.005
DBP, mmHg	82.67±11.43	84.02±10.29	85.84±9.48	88.75±8.68	0.002
BMI, kg/m2	23.34 (20.86, 25.73)	24.03 (21.48, 27.28)	25.8 (23.61, 27.34)	24.87 (23.43, 27.32)	0.003
MRC sum score	48 (30, 60)	48 (36, 58)	45 (36.75, 54.25)	42 (34.5, 55)	0.683
Cholesterol, mmol/L	3.57 (3.24, 4.16)	4.13 (3.62, 4.6)	3.91 (3.53, 4.47)	4.45 (4.05, 4.74)	<0.001
LDL, mmol/L	2.5 (1.88, 2.94)	2.6 (2.08, 3.14)	2.33 (1.86, 3.03)	2.46 (2.13, 2.87)	0.819
APOA1, g/L	1.15 (1.08, 1.34)	1.14 (1.01, 1.28)	1.15 (1.03, 1.31)	1.21 (1.07, 1.35	0.298
HDL, mmol/L	1.21 (1.04, 1.34)	1.12 (0.95, 1.25)	1.02 (0.94, 1.15)	1.04 (0.94, 1.15)	<0.001
Albumin, g/L	38.4 (35.6, 40.15)	37.5 (35.9, 39.55)	36.5 (34.65, 39.55)	37.7 (35.4, 40.3)	0.747
WBC count, 10^9/L	6.79 (4.77, 9.13)	6.25 (4.58, 7.71)	7.66 (6.28, 9.54)	7.34 (6.4, 8.91)	0.052
Platelet count, 10^9/L	257 (219, 305)	237 (188, 295.5)	239 (211.5, 302.5)	267 (215.5, 310.5)	0.573
Severe GBS, n (%)	27 (52.9)	24 (47.1)	37 (74.0)	39 (76.5)	0.001
Poor short-term prognosis, n (%)	19 (37.3)	13 (25.5)	18 (36.0)	26 (51.0)	0.088
Hypertension, n (%)	10 (19.6)	12 (23.5)	18 (36.0)	26 (51.0)	<0.001
Diabetes mellitus, n (%)	0 (0.0)	7 (13.7)	11 (22.0)	10 (19.6)	0.002
Heart disease, n (%)	0 (0.0)	2 (3.9)	2 (4.0)	4 (7.8)	0.053
Cigarette smoking, n (%)	13 (25.5)	7 (13.7)	16 (32.0)	19 (37.3)	0.055
Alcohol consumption, n (%)	15 (29.4)	8 (15.7)	13 (26.0)	13 (25.5)	0.956
Preceding infection, n (%)	38 (74.5)	33 (64.7)	30 (60.0)	33 (64.7)	0.251
Surgery, n (%)	8 (15.7)	6 (11.8)	12 (24.0)	5 (9.8)	0.927
Trauma, n (%)	5 (9.8)	8 (15.7)	12 (24.0)	7 (13.7)	0.502
Subtype					0.893
AIDP	26 (51.0)	24 (47.1)	26 (52.0)	27 (52.9)	
AMAN	9 (17.6)	12 (23.5)	14 (28.0)	12 (23.5)	
AMSAN	7 (13.7)	8 (15.7)	3 (6.0)	6 (11.8)	
MFS	9 (17.6)	7 (13.7)	7 (14.0)	6 (11.8)	

Data presented as mean ± SD, median (Q1-Q3) or percentage. GBS, Guillain-Barré syndrome; SBP, systolic blood pressure; DBP, diastolic blood pressure; BMI, Body mass index; MRC, Medical Research Council; TyG index, triglyceride-glucose index; LDL, low-density lipoprotein; APOA1, apolipoprotein A1; HDL, high-density lipoprotein; WBC, White Blood Cell; AIDP, acute inflammatory demyelinating polyneuropathy; AMAN, acute motor axonal neuropathy; AMSAN, acute motor sensory axonal neuropathy; MFS, Miller-Fisher syndrome.

### 3.5 ROC curve for predicting severe and poor short-term prognosis in GBS

**Figure 5:**
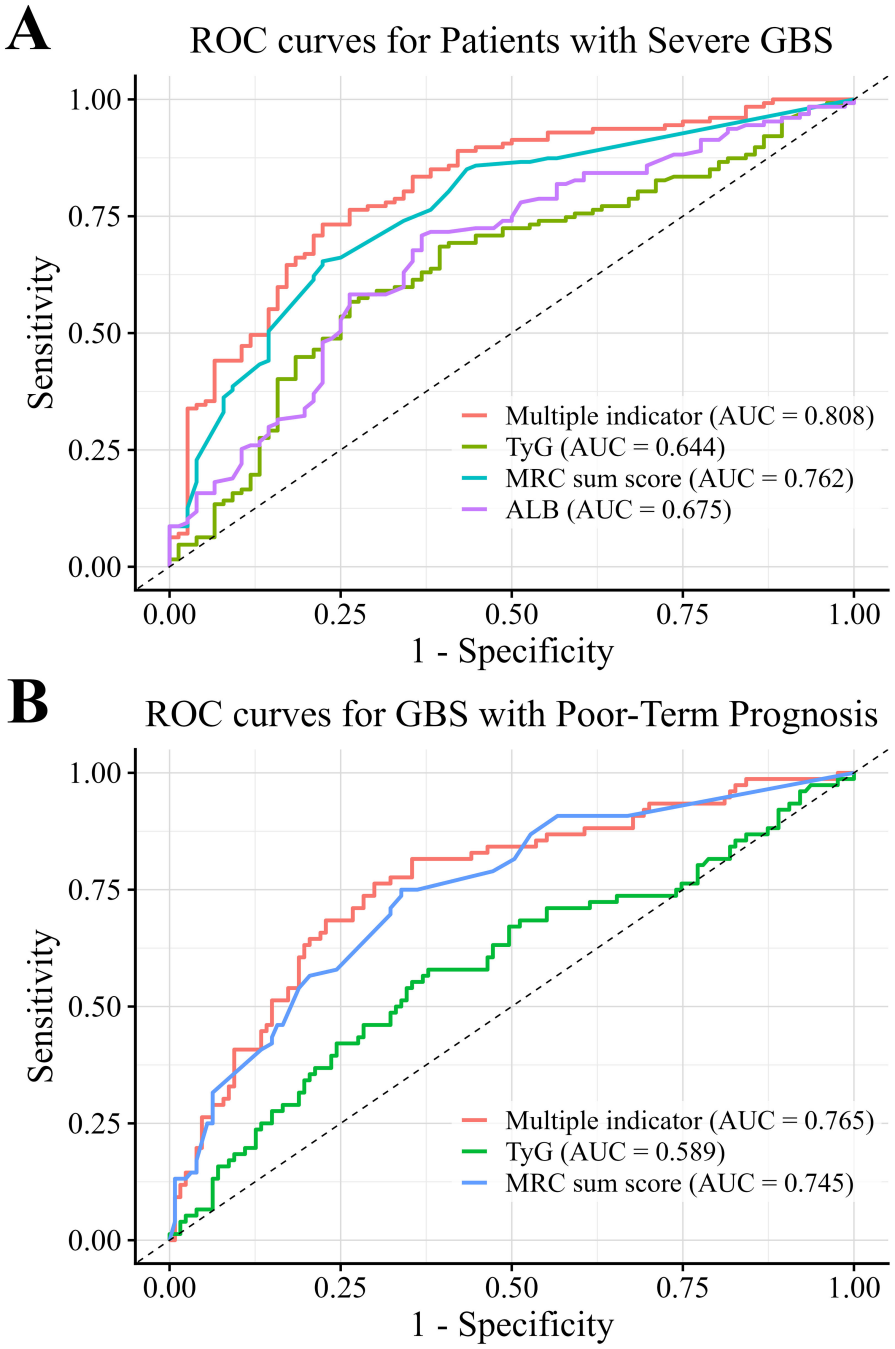
ROC curves of the TyG index and other traditional indices in relation to GBS. **(A)** The AUC values for the TyG index, MRC sum score, and ALB alone in predicting severe GBS were 0.644, 0.762, and 0.675, respectively, whereas that for the combined diagnostic approach increased to 0.808. **(B)** The AUC values for the TyG index and MRC sum score when predicting poor short-term prognosis in GBS were 0.589 and 0.745, respectively, whereas that for the integrated diagnostic approach increased to 0.765.

ROC curve analysis (**Figure 5**; **Table 5**) was performed to predict severe GBS. The optimal cutoff value of the TyG index was 8.67, corresponding to an AUC of 0.644, a sensitivity of 0.567, and a specificity of 0.737. The optimal cutoff of the MRC sum score was 45.5 (AUC = 0.762, sensitivity = 0.654, specificity = 0.776), and the optimal cutoff of serum ALB was 38.45 (AUC = 0.675, sensitivity = 0.709, specificity = 0.632). For the combined multi-index predictive model, the cutoff value was 0.63, with an AUC of 0.808, a sensitivity of 0.732, and a specificity of 0.776. DeLong test further confirmed that the combined model exhibited significantly better predictive performance than any single indicator alone (all P<0.05).

**Table 5:** ROC analysis of different indicators in patients with mild or severe GBS.

Indicator	Cut-off	AUC	*P* value	95% CI		Predict	Sensitivity	Specificity	Delong test with multiple indicator	
				Lower	Upper				Z value	*P* value
Multiple indicator	0.63	0.808		0.746	0.87		0.732	0.776		
TyG	8.67	0.644	0.001	0.565	0.723	Positive	0.567	0.737	3.837	<0.001
MRC sum score	45.5	0.762	<0.001	0.695	0.829	Negative	0.654	0.776	2.207	0.027
ALB	38.45	0.675	0.006	0.598	0.752	Negative	0.709	0.632	3.765	<0.001

For the prediction of short-term adverse prognosis in GBS patients (**Figure 5**; **Table 6**), the optimal cutoff of the TyG index was also 8.67 (AUC = 0.589, sensitivity = 0.579, specificity = 0.622), and the cutoff of the MRC sum score remained 45.5 (AUC = 0.745, sensitivity = 0.750, specificity = 0.661). The combined multi-index model achieved a cutoff of 0.36, with an AUC of 0.765, a sensitivity of 0.763, and a specificity of 0.701. DeLong test showed that the predictive efficacy of the combined model was significantly superior to the TyG index alone (P<0.05), whereas no significant difference was found between the combined model and the MRC sum score (P = 0.152).

**Table 6:** ROC analysis of different indicators in GBS with good or poor short-term prognosis.

Indicator	Cut-off	AUC	*P* value	95% CI		Predict	Sensitivity	Specificity	Delong test with multiple indicator	
				Lower	Upper				Z value	*P* value
Multiple indicator	0.36	0.765		0.696	0.833		0.763	0.701		
TyG	8.67	0.589	0.036	0.506	0.672	Positive	0.579	0.622	3.718	<0.001
MRC sum score	45.5	0.745	<0.001	0.675	0.814	Negative	0.750	0.661	1.431	0.152

Bootstrap resampling was applied for internal validation to assess model overfitting and robustness. For the severe GBS prediction model, the apparent AUC was 0.808, with an optimism of 0.008 and a corrected AUC of 0.800. For the model predicting poor short-term prognosis of GBS, the apparent AUC was 0.765, with an optimism of 0.006 and a corrected AUC of 0.759. The low optimism magnitude and well-maintained corrected AUCs suggested minimal overfitting, indicating favorable stability and reliability of the established models (**Supplementary Table 2**).

Furthermore, incremental predictive value of the TyG index was evaluated by NRI and IDI. When the TyG index was incorporated into the baseline model for severe GBS prediction, the NRI was 0.534 (95% CI: 0.263–0.804, P<0.001) and the IDI was 0.047 (95% CI: 0.017–0.077, P=0.002). For patients with short-term poor prognosis of GBS, the NRI was 0.402 (95% CI: 0.123–0.681, P = 0.005) and the IDI was 0.022 (95% CI: 0.001–0.043, P = 0.039) (**Supplementary Table 3**). These findings demonstrated that the TyG index achieved a notable improvement in risk reclassification, while the integrated discrimination gain was modest for predicting severe GBS and short-term adverse prognosis.

## 4 Discussion

This study is the first to systematically explore the relationships between the TyG index and disease severity as well as short-term prognosis in patients with GBS. After rigorous variable screening and adjustment for confounding factors, regression analysis confirmed that the TyG index serves as an independent risk factor for severe GBS, whereas ALB and the MRC sum score are independent protective factors. RCS analysis further demonstrated a linear dose–response association between the TyG index and the risk of severe GBS. In terms of short-term prognosis, the TyG index also emerged as an independent risk factor for adverse clinical outcomes, while the MRC sum score served as a protective factor. RCS analysis further verified a linear correlation between the TyG index and unfavorable short-term prognosis. Predictive validation indicated that incorporating the TyG index into the baseline model yielded a substantial increase in NRI, which significantly enhanced risk stratification and reclassification performance for both severe GBS and short-term adverse prognosis. By contrast, IDI only showed a marginal improvement, suggesting a limited incremental effect on overall predictive calibration.

At present, research on the association between the TyG index and nervous system demyelinating diseases remains limited. Specifically, no studies worldwide have investigated the relationships of the TyG index with disease severity and short-term prognosis in patients with GBS, resulting in a lack of available evidence for cross-study comparison. Relevant data on central nervous system demyelinating disorders are also scarce; only one study has reported a genetic association between the TyG index and an increased risk of multiple sclerosis (26). In contrast, the TyG index has been well established as an independent predictor of adverse clinical outcomes in cardiovascular, cerebrovascular, infectious and other chronic diseases, though obvious heterogeneity in OR estimates exists across different cohorts. Published studies have reported conflicting results regarding the association pattern between the TyG index and disease risk. Some studies have demonstrated a linear correlation consistent with our findings (27–29), whereas others have indicated a nonlinear relationship (30, 31).

With increasing TyG index quartiles, the risks of severe GBS and unfavorable short-term prognosis showed an overall upward trend, whereas pairwise comparisons between some adjacent quartile subgroups did not achieve statistical significance. Although a prominent inflection point was visually identified in the RCS curve, the nonlinear association failed to reach statistical significance. Intriguingly, the apparent inflection point from our RCS analysis showed a high numerical agreement with existing TyG cut-offs of 8.82 for 28-day mortality in critically ill septic ICU patients (30) and 8.80 for major adverse cardiovascular and cerebrovascular events in coronary artery disease patients (31). This numerical similarity further indicates that elevated TyG levels are correlated with a higher risk of adverse clinical outcomes in high-risk populations.

Elevated serum ALB levels were significantly negatively correlated with the risk of severe GBS and unfavorable short-term prognosis, which is consistent with previous research findings (32, 33). Willem-Jan R. Fokkink et al. observed that GBS patients may develop hypoalbuminemia after IVIg treatment, and this condition may be associated with more severe clinical manifestations and poor prognosis (32). Additionally, other studies have indicated that serum ALB levels tend to decrease during the subacute phase of GBS, and there may be a negative correlation between albumin concentrations and Hughes scores at both admission and discharge (34). As a negative acute-phase protein, ALB may reflect the body’s inflammatory status; hypoalbuminemia may therefore be associated with changes in other acute-phase reactants and elevated CRP levels (35, 36). Pro-inflammatory factors may widen endothelial gaps, leading to ALB extravasation and reduced circulating ALB levels, which may be linked to axonal injury. Furthermore, ALB may play a role in maintaining colloid osmotic pressure; insufficient ALB levels may result in inadequate endoneurial microcirculation, further affecting disease severity (37, 38).

The present study indicated that an elevated admission MRC sum score correlated with a lower risk of severe GBS and short-term adverse prognostic outcomes. These results aligned with findings from van Doorn et al. that a low MRC sum score at admission and at one week was independently linked to an inability to walk at 4 weeks, 3 months, and 6 months after the onset of GBS (39). The MRC scale is a standardized clinical evaluation tool extensively utilized to assess muscle strength and gauge the severity of neuromuscular disorders. The MRC sum score can facilitate the early identification of high-risk cases and optimize resource allocation, but should be interpreted with caution. This association should not be construed as a causal relationship; thus, future multicenter studies with expanded sample sizes are imperative to further investigate the predictive utility of the MRC sum score in GBS patients.

In patients with severe GBS, quartile analysis showed that the Q2 group had a slightly higher but non-significant risk of severe illness compared with the Q1 group, while the Q3 and Q4 groups had a significantly higher risk. This indicates that elevated TyG levels produce an obvious cumulative adverse effect on the development of severe GBS. The RCS curve showed that disease risk rose rapidly at high TyG levels with a visible inflection point, but the nonlinear trend was not statistically significant, and the overall association remained linear. The inconsistent findings between quartile stratification and RCS analysis are mainly due to differences in statistical methods. Quartile grouping converts continuous variables into categorical subgroups and easily enlarges the risk gap between extreme groups. In contrast, RCS uses continuous raw data, adjusts for confounding factors more adequately, and adopts stricter statistical standards; minor local changes in the curve do not change the overall linear trend.

For short-term poor prognosis, multivariate logistic regression showed that elevated TyG index was an independent risk factor, with a borderline significant P value of 0.068. This result can be explained in two aspects. Statistically, logistic regression has higher statistical power for overall association testing, whereas RCS spline fitting uses more rigorous adjustment and conservative testing, making it less likely to obtain significant results. Clinically, short-term neurological recovery in GBS patients is affected by respiratory complications, immunotherapy effect and acute autonomic dysfunction (40, 41), which weakens the correlation between baseline glycolipid metabolic markers and short-term prognosis.

Quartile stratification showed that the risks of severe GBS and poor short-term prognosis gradually increased with rising TyG levels, but some pairwise comparisons between adjacent quartiles were not statistically significant. Significant differences among subgroups were only observed for severe GBS in high TyG strata, while no quartile comparison reached significance for short-term prognosis. RCS based on continuous data can sensitively capture subtle rising cumulative trends. In comparison, quartile stratification tends to lower the statistical power of pairwise comparisons owing to sample partitioning, narrow numerical intervals between adjacent quantiles, and adjustment for multiple comparisons. In conclusion, although quartile stratification, RCS fitting and logistic regression showed inconsistent statistical significance, all methods reflected the same biological trend. Elevated TyG index is consistently associated with higher risks of severe GBS and unfavorable short-term prognosis, and there is no logical contradiction among the results.

Two predictive models were established in this study to identify severe illness status and unfavorable short-term prognosis in patients with GBS. ROC curves were used to compare the early discriminatory efficiency of single indicators and combined multi-indicator models. In both models, the AUC of each individual biomarker was generally unsatisfactory, with room for improvement in sensitivity and specificity. Compared with single traditional indicators, the combined model exhibited superior overall discriminatory performance, accompanied by increases in sensitivity, specificity and AUC. After incorporating the TyG index, the NRI of the model increased significantly, and the risk reclassification efficiency for severe illness and unfavorable short-term prognosis was substantially enhanced. Although the IDI only showed a slight elevation, both differences were statistically significant. NRI mainly reflects the model’s ability to correct individual case reclassification based on clinical risk thresholds. A significant increase in NRI indicated that the TyG index could effectively optimize the accuracy of population risk stratification. IDI focuses on evaluating the discriminatory separation efficiency of the overall predicted probability of the model, and its slight rise suggested that the TyG index exerted a relatively limited effect on improving the global probability discrimination between event and non-event groups. Overall, the incremental benefit of the TyG index for the predictive model was mainly reflected in the improvement of risk reclassification performance, while its enhancement on the overall discriminatory efficiency of the model was relatively modest.

Notably, after adding the TyG index in this study, no obvious improvements were observed in AUC, sensitivity or specificity; nevertheless, statistically significant differences were still found in NRI and IDI. This phenomenon is reasonable in incremental research of clinical prediction models. AUC, sensitivity and specificity mainly reflect the overall discriminatory capability and classification efficiency under fixed cut-off values, and are insensitive to subtle local changes in individual risk stratification. In contrast, NRI and IDI can sensitively capture the subtle gains in individual risk reclassification optimization and predicted probability separation brought by novel biomarkers. Therefore, the pattern of modest improvement in overall diagnostic efficiency with statistically significant benefits in reclassification indicators is consistent with the statistical principles for incremental evaluation of prediction models.

## 5 Strengths and limitations

This study presents several notable strengths. Firstly, GBS is a rare neurological disorder, and with a total of 203 cases included, the sample size is adequate for analysis. Secondly, this research is pioneering in examining the relationship of the TyG index with the risk of severe GBS and short-term poor prognosis in affected patients. Furthermore, it makes the first attempt to assess the diagnostic efficacy of the TyG index in identifying severe GBS and predicting poor short-term prognosis, both in isolation and in combination with traditional indicators. Nonetheless, the study also has certain limitations. Firstly, this study employed a retrospective cohort design that relied on pre-existing clinical data for analysis. Retrospective studies are inherently vulnerable to selection and information biases; therefore, the associations identified in this study should be considered descriptive rather than causal. Then, although we adjusted for known confounders such as age, sex, and comorbidities in our analysis, the retrospective nature of the study limits our ability to account for other unmeasured confounding factors. For instance, variables such as lifestyle, dietary habits, and psychological stress were not included in our analysis, yet they may significantly influence GBS prognosis and introduce potential bias into our results. Consequently, these unmeasured confounding factors may affect the observed relationship between the TyG index and short-term outcomes. Additionally, this investigation is based solely on findings from a single center, warranting further validation across multiple centers and diverse populations.

## 6 Conclusions

The TyG index is established as an independent risk factor for severe GBS and for GBS associated with poor short-term prognosis. Notably, the TyG index exhibits a linear relationship with both severe GBS and GBS with poor short-term prognosis. Furthermore, when used in combination with conventional indicators, the TyG index demonstrates certain diagnostic value in identifying severe GBS and predicting short-term poor prognosis. However, given the limitations of retrospective data, these findings require further validation in larger, rigorously designed prospective multicenter studies.

## Data Availability

The original contributions presented in the study are included in the article/**Supplementary Material**. Further inquiries can be directed to the corresponding authors.
