# Supplementary material for: Association of the triglyceride-glucose index with the severity and short-term prognosis of Guillain-Barré syndrome
Source: Front Immunol. 2026 Jun 8;17:1813152. doi: 10.3389/fimmu.2026.1813152 (PMC13283979; doi:10.3389/fimmu.2026.1813152)
Supplement: Supplementary file 1 [file SupplementaryFile1.docx]

Supplement Table 1: Assessment of multicollinearity using VIF in logistic regression models

| Model | Variable | VIF |
| --- | --- | --- |
| Severe GBS | MRC sum score | 1.024702 |
|  | Albumin | 1.024702 |
|  | TyG index | 1.025668 |
| GBS with Poor short-term prognosis | MRC sum score | 1.021783 |
|  | TyG index | 1.021783 |

VIF: variance inflation factor

Supplement Table 2. Bootstrap validation of model performance

| Model | Apparent AUC | Optimism bias | Optimism corrected AUC |
| --- | --- | --- | --- |
| Severe GBS | 0.808 | 0.008 | 0.800 |
| Poor short-term GBS | 0.765 | 0.006 | 0.759 |

Supplement Table 3. Incremental predictive value assessed by NRI and IDI

| Outcome | Model comparison | NRI (95%CI) | P value | IDI (95%CI) | P value |
| --- | --- | --- | --- | --- | --- |
| Severe GBS | MRC + ALB  MRC +ALB + TyG | 0.534 (0.263–0.804) | <0.001 | 0.047 (0.017–0.077) | 0.002 |
| Poor short-term GBS | MRC  MRC + TyG | 0.402 (0.123–0.681) | 0.005 | 0.022 (0.001–0.043) | 0.039 |
